# Advanced MRI, Radiomics and Radiogenomics in Unravelling Incidental Glioma Grading and Genetic Status: Where Are We?

**DOI:** 10.3390/medicina61081453

**Published:** 2025-08-12

**Authors:** Alessia Guarnera, Tamara Ius, Andrea Romano, Daniele Bagatto, Luca Denaro, Denis Aiudi, Maurizio Iacoangeli, Mauro Palmieri, Alessandro Frati, Antonio Santoro, Alessandro Bozzao

**Affiliations:** 1 Neuroradiology Unit, NESMOS (Neuroscience, Mental Health and Sensory Organs) Department, Sant’Andrea Hospital, La Sapienza University, Via di Grottarossa, 1035-1039, 00189 Rome, Italy; guarneraalessia@gmail.com (A.G.); alessandro.bozzao@uniroma1.it (A.B.); 2 Academic Neurosurgery, Department of Neurosciences, University of Padova, 35128 Padova, Italy; tamara.ius@gmail.com (T.I.); luca.denaro@unipd.it (L.D.); 3 Neuroradiology Unit, Department of Diagnostic Imaging, University Hospital of Udine, Piazzale Santa Maria della Misericordia 15, 33100 Udine, Italy; daniele.bagatto@gmail.com; 4 Neurosurgical Department, Marche Polytechnic University, 60121 Ancona, Italy; denis.aiudi@gmail.com (D.A.); m.iacoangeli@univpm.it (M.I.); 5 Human Neurosciences Department, Neurosurgery Division, Sapienza University, 00185 Rome, Italy; mauro.palmieri@uniroma1.it (M.P.); alex.frati@gmail.com (A.F.); antonio.santoro@uniroma1.it (A.S.)

**Keywords:** radiomics, radiogenomics, incidental gliomas, low-grade glioma, high-grade glioma, PWI, DTI, IDH, MGMT, EGFR, personalised medicine

## Abstract

The 2021 WHO classification of brain tumours revolutionised the oncological field by emphasising the role of molecular, genetic and pathogenetic advances in classifying brain tumours. In this context, incidental gliomas have been increasingly identified due to the widespread performance of standard and advanced MRI sequences and represent a diagnostic and therapeutic challenge. The impactful decision to perform a surgical procedure deeply relies on the non-invasive identification of features or parameters that may correlate with brain tumour genetic profile and grading. Therefore, it is paramount to reach an early and proper diagnosis through neuroradiological techniques, such as MRI. Standard MRI sequences are the cornerstone of diagnosis, while consolidated and emerging roles have been awarded to advanced sequences such as Diffusion-Weighted Imaging/Apparent Diffusion Coefficient (DWI/ADC), Perfusion-Weighted Imaging (PWI), Magnetic Resonance Spectroscopy (MRS), Diffusion Tensor Imaging (DTI) and functional MRI (fMRI). The current novelty relies on the application of AI in brain neuro-oncology, mainly based on radiomics and radiogenomics models, which enhance standard and advanced MRI sequences in predicting glioma genetic status by identifying the mutation of multiple key biomarkers deeply impacting patients’ diagnosis, prognosis and treatment, such as IDH, EGFR, TERT, MGMT promoter, p53, H3-K27M, ATRX, Ki67 and 1p19. AI-driven models demonstrated high accuracy in glioma detection, grading, prognostication, and pre-surgical planning and appear to be a promising frontier in the neuroradiological field. On the other hand, standardisation challenges in image acquisition, segmentation and feature extraction variability, data scarcity and single-omics analysis, model reproducibility and generalizability, the black box nature and interpretability concerns, as well as ethical and privacy challenges remain key issues to address. Future directions, rooted in enhanced standardisation and multi-institutional validation, advancements in multi-omics integration, and explainable AI and federated learning, may effectively overcome these challenges and promote efficient AI-based models in glioma management. The aims of our multidisciplinary review are to: (1) extensively present the role of standard and advanced MRI sequences in the differential diagnosis of iLGGs as compared to HGGs (High-Grade Gliomas); (2) give an overview of the current and main applications of AI tools in the differential diagnosis of iLGGs as compared to HGGs (High-Grade Gliomas); (3) show the role of MRI, radiomics and radiogenomics in unravelling glioma genetic profiles. Standard and advanced MRI, radiomics and radiogenomics are key to unveiling the grading and genetic profile of gliomas and supporting the pre-operative planning, with significant impact on patients’ differential diagnosis, prognosis prediction and treatment strategies. Today, neuroradiologists are called to efficiently use AI tools for the in vivo, non-invasive, and comprehensive assessment of gliomas in the path towards patients’ personalised medicine.

## 1. Introduction

Incidental Low-Grade Gliomas (iLGGs) are rare tumoural entities that are incidentally detected at imaging in asymptomatic patients with an incidence of 0.04% to 0.2% in the general asymptomatic population. Traditionally considered benign, iLGGs are now recognised as evolving and progressive lesions, albeit at a slower pace compared to symptomatic Low-Grade Gliomas (LGGs). The proper and early diagnosis of iLGGs, which has been increasingly made thanks to the widespread performance of standard and advanced MRI sequences, has crucial implications for patients’ prognosis, treatment choice and quality of life [1].

The first impactful decision is between early intervention and a strategy of watchful waiting, which is based on the tumour grading, location and patients’ comorbidities. Particularly, iLGGs located in non-surgical areas, such as the basal ganglia, require a traditional conservative strategy, known as “wait and see” [2,3,4]. In resectable tumours, early surgery leads to a greater extent of resection, improved return-to-work timing, overall survival and progression-free survival [2,3,5,6]

The 2021 WHO classification of brain tumours revolutionised the oncological field by emphasising the role of molecular, genetic and pathogenetic advances in classifying brain tumours [7]. The decision to perform a surgical procedure also relies on the non-invasive identification of features or parameters that may correlate with brain tumour genetic profile and grading [8,9].

Brain MRI standards, such as TWI (weighted image), T2WI and Fluid-Attenuated Inversion Recovery (FLAIR), and advanced sequences may guide the differential diagnosis and pre-surgical planning of LGGs as compared to High-Grade Gliomas (HGG) and demonstrated [10]. Consolidated and emerging roles have been awarded to sequences such as Diffusion-Weighted Imaging/Apparent Diffusion Coefficient (DWI/ADC), which gives crucial insights into tumour cellularity, and Perfusion-Weighted Imaging (PWI), which provides qualitative and quantitative information on tumoural vascularity and perfusion, and Magnetic Resonance Spectroscopy (MRS), which evaluates the metabolites within the lesion without performing biopsies [8,11]. Moreover, advanced techniques such as Diffusion Tensor Imaging (DTI) and functional MRI (fMRI) support the differential diagnosis of iLGGs and also play a critical role in the pre-surgical planning with the balanced aims of radical resections and preserving functions. fMRI allows for the precise mapping of eloquent brain areas, such as those responsible for motor and language functions, while DTI allows the visualisation of white matter tracts in relation to the tumour [12,13,14].

Moreover, the integration of MRI images into AI-based radiomics and radiogenomics models has demonstrated accuracy in glioma detection, grading, prognostication, and pre-surgical planning, as well as in the prediction of glioma genetic status. In fact, the identification of key biomarker mutations, such as IDH, EGFR, TERT, MGMT promoter, p53, H3-K27M, ATRX, Ki67 and 1p19q, carries a significant impact on the diagnosis, prognosis and treatment as well as on patients’ quality of life. Early genetic profiling is critical because it guides a tailored therapy of brain glioma and supports patient-oriented personalised medicine [9,15,16,17,18].

Current literature mainly focuses on the roles of MRI and AI models in glioma diagnosis, prognosis and management separately, while the integrated presentation of MRI, radiomics and radiogenomics advancements in the field, with a specific and extensive analysis of their efficiency in glioma key biomarker mutations [8,9,10,11,12,13,14,15,16,17].

Our multidisciplinary review aims to bridge this gap by: (1) extensively presenting the role of standard and advanced MRI sequences in the differential diagnosis of iLGGs as compared to HGGs (High-Grade Gliomas); (2) giving an overview of the current and main applications of AI tools in the differential diagnosis of iLGGs as compared to HGGs; (3) showing the role of MRI, radiomics and radiogenomics in unravelling glioma genetic profile. 

Therefore, we organised the review in the following paragraphs: The Role of MRI in Glioma Detection and Grading; The Role of AI in Glioma Detection, Grading and Prediction of Genetic Profile; and The Role of MRI, Radiomics and Radiogenomics in Unravelling Glioma Genetic Profile.

## 2. The Role of MRI in Glioma Detection and Grading

Since incidentalomas are asymptomatic tumours, it is paramount to obtain a certain diagnosis and choose the proper management strategy based on patients’ characteristics, lesion features, topography, and grade. MRI plays a pivotal role in this field, and the optimal protocol includes the standard sequences and advanced sequences that are key to identifying, characterising, and grading the tumour [14] (Table 1).

### 2.1. Standard MRI Sequences

**Figure 1:**
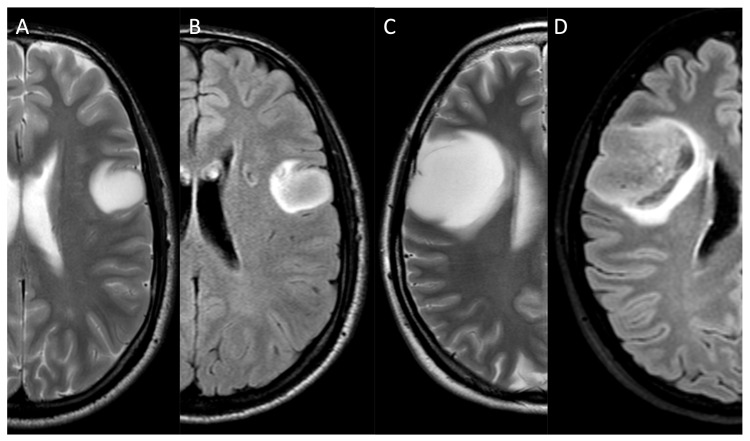
Shows the brain MRIs of two patients affected by LGGs. A 32-year-old man affected by an incidental grade 2, IDH-mutant diffuse astrocytoma involving the inferior and lateral (fronto-opercular) portion of the left frontal lobe. Axial T2WI (**A**) demonstrate a well-circumscribed hyperintense lesion, with the typical T2WI/FLAIR (**A**,**B**) mismatch. A 31-year-old woman affected by an incidental grade 2, IDH-mutant diffuse astrocytoma involving the fronto-opercular portion of the right frontal lobe (**C**,**D**). Axial T2WI (**C**) and FLAIR (**D**) sequences show a round lesion with the typical T2/FLAIR mismatch sign. *LGG: low-grade glioma; WI: weighted image; FLAIR: fluid-attenuated inversion recovery*.

Standard brain MRI sequences include T1-weighted image (T1WI), T2-weighted image (T2WI), and Fluid-Attenuated Inversion Recovery (FLAIR), which form the cornerstone of initial neuroimaging evaluation and play a crucial role in the detection, characterisation, and localisation of iLGGs [19,20]. iLGGs, which are commonly located in the fronto-temporal lobes, generally appear as hypointense lesions on T1WI and hyperintense on T2WI and FLAIR, reflecting the increased tissue water content and altered ultrastructure characteristic of gliomas (Figure 1).

**Figure 2:**
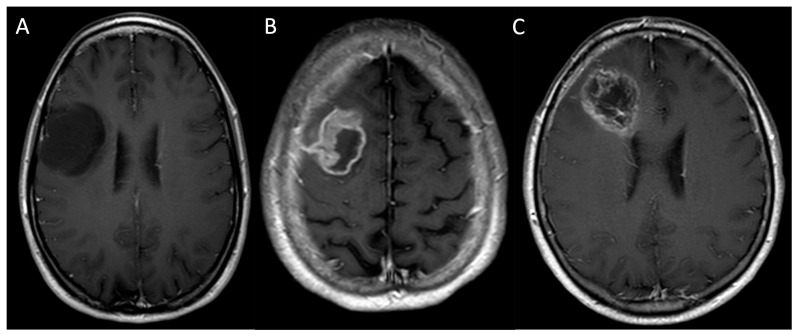
Compares the post-contrast T1WI sequences of patients affected by a grade 2, IDH-mutant diffuse astrocytoma involving the fronto-opercular portion of the right frontal lobe (**A**), a grade 4, IDH wild-type primary glioblastoma of the premotor area of the right frontal lobe (**B**), and a grade 4, IDH wild-type primary glioblastoma of the right frontal lobe. The LGG (**A**) appears hypointense with no contrast enhancement, while the HGGs (**B**,**C**) appear as inhomogeneous lesions with strong and irregular “ring-enhancement” surrounding the necrotic core (**B**,**C**). *LGG: low-grade glioma; HGG: high-grade glioma; WI: weighted image*.

The FLAIR sequence is particularly valuable as it suppresses the signal from cerebrospinal fluid, allowing for a clearer delineation of the non-enhancing tumour component, a hallmark of many LGGs [14]. Interestingly, IDH-mutant, 1p19q non-codeleted astrocytomas, which are frequently located in the frontal and temporal lobes, are characterized by “T2-FLAIR mismatch”, namely a different appearance of the lesion between the two sequences characterized by T2 hyperintensity and incomplete attenuation on FLAIR, defined by a core inhomogeneous hypointensity surrounded by a peripheral rim of hyperintensity (Figure 1). This sign presents a low sensitivity and high specificity for differentiating low- and high-grade lesions [21]. Recently, Li et al. described the wave-like intratumoral-wall (SWITW) sign on T2WI, namely the presence of a wave-like intertumoral wall embracing a T2 hyperintense tumour core. This sign represents a sensitive and specific biomarker to diagnose IDH-mutant 1p/19q codeleted oligodendrogliomas, which benefit from a conservative surgery and show a good response to chemotherapy and radiotherapy and an improved outcome [22]. T1WI without contrast supports the identification of blood products, fat and melanin that can guide the differential diagnosis. In fact, intratumoral haemorrhage is characteristic of HGGs as compared to LGGs [10]. Contrast enhancement, assessed with gadolinium-based contrast agents on T1WI, is usually absent or rarely appears as patchy areas, suggesting a lower-grade tumour with an intact or minimally disrupted blood–brain barrier (Figure 2A) [14]. Intense and inhomogeneous contrast enhancement is typical of HGGs and testifies to the disruption of the brain blood barrier (Figure 2B,C).

**Figure 3:**
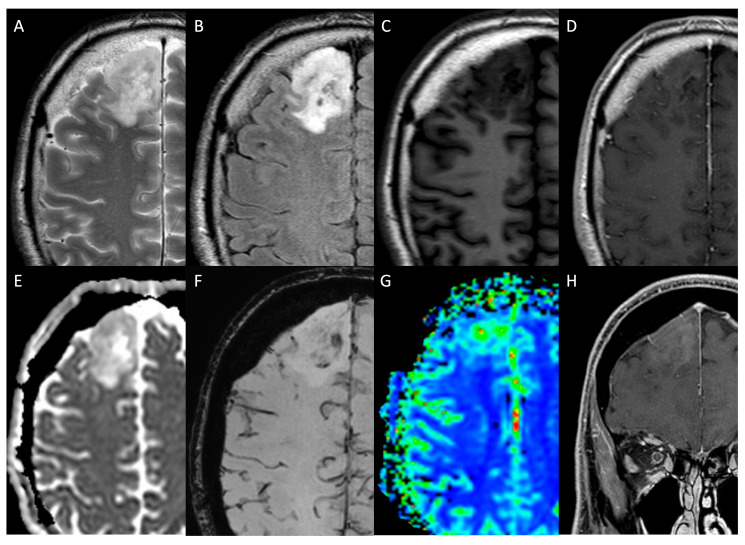
A 48-year-old woman affected by histologically proven grade 2, IDH-mutant, 1p/19q codeleted oligodendroglioma centred in the right superior frontal gyrus and appearing heterogeneously hyperintense on axial T2WI and FLAIR sequences (**A**,**B**) and hypointense on T1WI (**C**) with poor/absent enhancement on post-contrast axial (**D**) and coronal fat-saturated T1WI (**H**). On DSC-PWI (**G**), the lesion is characterised by high rCBV, while there is an intermediate signal on ADC (**E**) and some “blooming” foci on SWI (**F**). *WI: weighted image; FLAIR: fluid-attenuated inversion recovery; DSC: dynamic susceptibility contrast; PWI: perfusion WI; rCBV: relative cerebral blood volume; ADC: apparent diffusion coefficient; SWI: susceptibility weighted image*.

On the other hand, there are two crucial exceptions, the first being the IDH-mutant and 1p/19q codeleted oligodendrogliomas, which are commonly located in the frontal lobes and are highly heterogeneous with frequent calcifications and present a variable degree of contrast enhancement (Figure 3) [8].

**Figure 4:**
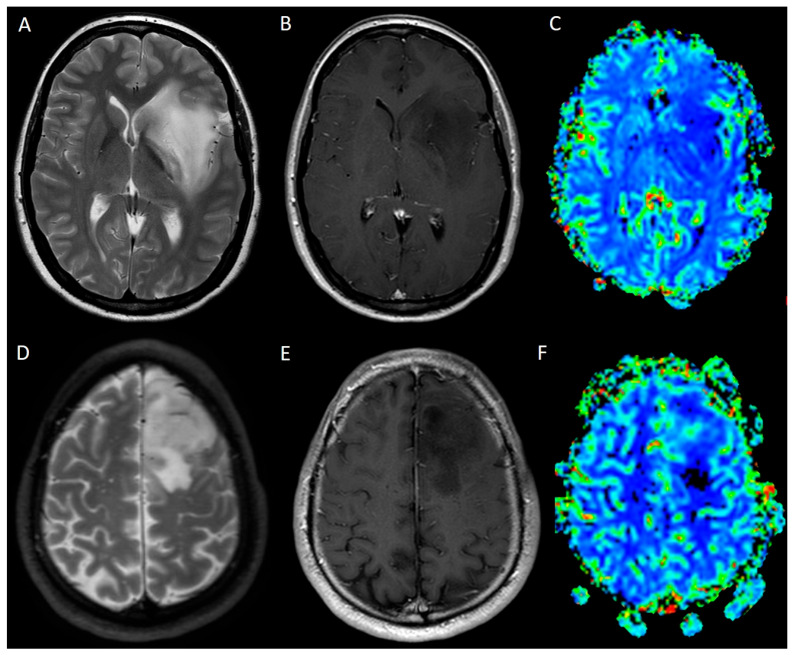
Comparison between a large grade 2 IDH-mutant glioma involving the left insular region (**A**–**C**) and a grade 4 IDH wild-type glioma involving the left frontal lobe (**D**–**F**). Both lesions appear heterogeneously hyperintense on T2WI (**A**,**D**) with no significant contrast enhancement on T1WI (**B**,**E**). Nevertheless, PWI-DSC rCBV suggests the differential diagnosis based on the increased perfusion identifiable in the grade 4 IDH wild-type glioma (**F**) as compared to the absence of increased perfusion of the grade 2 IDH-mutant glioma (**C**). *WI: weighted image; DSC: dynamic susceptibility contrast; PWI: perfusion WI; rCBV: relative cerebral blood volume*.

The second one refers to poorly enhancing HGGs, namely HGGs, which do not show significant contrast enhancement, particularly in their early stages or in certain histological subtypes, and whose neoangiogenesis may mainly be identified thanks to increased perfusion on PWI (Figure 4) [14].

**Figure 5:**
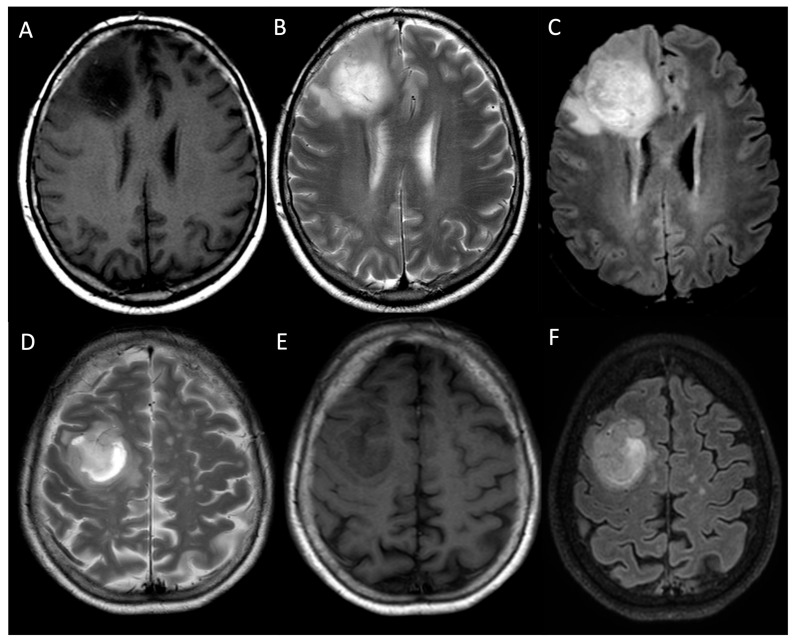
Shows a grade 4, IDH wild-type primary glioblastoma of the right frontal lobe (**A**–**C**) and a grade 4, IDH wild-type primary glioblastoma of the premotor area of the right frontal lobe (**D**–**F**). Both lesions appear inhomogeneously hyperintense on T2WI (**B**,**D**) and FLAIR (**C**,**F**), and inhomogeneously hypointense on T1WI (**A**,**E**). Peripheral vasogenic oedema appears hypointense on T1WI (**A**,**E**) and hyperintense on T2WI/FLAIR (**B**–**D**,**F**). *WI: weighted image; FLAIR: fluid-attenuated inversion recovery*.

Finally, gliomas are usually surrounded by peritumoral oedema, appearing hyperintense on T2WI/FLAIR and causing mass effect on adjacent structures such as cisterns or ventricles, sometimes scarcely distinguishable from the tumour itself. This oedema may have a vasogenic origin, represented by an increase in extracellular water caused by plasma leakage, or an infiltrative nature. The latter is typical of HGG and cannot be easily separated by vasogenic oedema except with advanced sequences such as DWI, DTI, MRS and PWI sequences (Figure 5) [23].

### 2.2. Advanced MRI Sequences

Despite their fundamental role in detecting iLGGs, standard MRI sequences have inherent limitations in the differential diagnosis with HGGs and pre-surgical planning since HGGs may show a similar presentation on standard MRI sequences as compared to LGGs (Figure 5). Furthermore, monitoring disease progression of LGGs to HGGs can be particularly difficult due to their characteristically slow growth rates and anisotropic patterns of infiltration [20]. This diagnostic ambiguity underscores the need for advanced imaging techniques to provide more specific information about the underlying tumour biology and genetics and aid in accurate glioma grading and risk assessment [14].

#### 2.2.1. Black-Blood Imaging

Post-contrast black-blood (BB) MRI sequence has been awarded an emerging role in improving brain tumour detection and characterisation, vascular architecture delineation and treatment planning [24]. Particularly, BB imaging effectively suppresses flowing blood signals and enhances the visualisation of vessel walls and perivascular spaces. HGGs, characterised by aggressive angiogenesis and abnormal vascularity, often demonstrate irregular and tortuous vessel morphology, intralesional haemorrhages, and increased vessel wall thickening. Conversely, LGGs typically exhibit less vascular proliferation and more regular vessel structures, leading to subtle or absent vessel wall abnormalities. Finck et al. demonstrated that BB imaging considerably improves the delineation of therapy-naive HGGs by offering a better definition of tumour boundaries and spread of HGGs into adjacent parenchyma, which are crucial parameters in pre-surgical planning [24]. Moreover, Kammer et al. showed that the 3D T1 TSE black-blood sequence with sub-millimetre resolution (T1-mVISTA) improves detection rates of small lesions in the disease’s early stages as compared to 3D T1 MPRAGE [25], pairing the findings of Oh et al., who suggested that BB imaging improves the diagnostic accuracy and predicts progression-free survival in patients with leptomeningeal carcinomatosis [26]. Although BB Imaging is not routinely performed in clinical routine, it represents a valuable sequence to be included in MRI protocols for brain tumours.

#### 2.2.2. Perfusion-Weighted Imaging

Perfusion-Weighted Imaging (PWI) encompasses a range of advanced MRI techniques, including Dynamic Susceptibility Contrast (DSC), Dynamic Contrast Enhancement (DCE), and Arterial Spin Labelling (ASL), which are designed to evaluate the microvasculature and blood flow characteristics within and in the adjacent tissue surrounding brain tumours. Since tumour vascularity usually correlates with glioma grade, PWI plays a significant role in differentiating iLGGs from HGGs based on their distinct vascular profiles [8,27]. The differential diagnosis guides pre-operative risk assessment and treatment decisions in patients with iLGGs who are currently asymptomatic [9,27].

#### Dynamic Susceptibility Contrast

**Figure 6:**
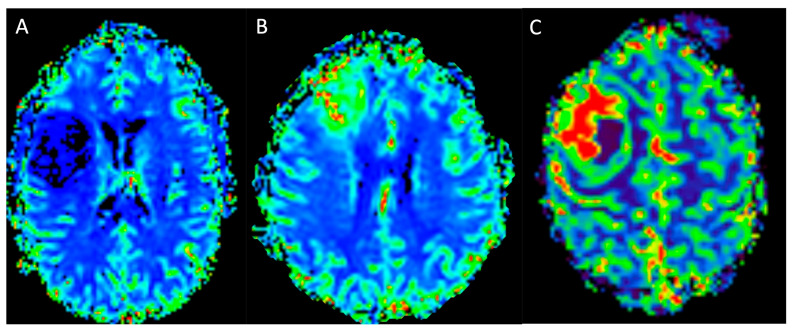
Compares the PWI-DSC-derived rCBV maps of patients affected by a grade 2, IDH-mutant diffuse astrocytoma involving the fronto-opercular portion of the right frontal lobe (**A**), a grade 4, IDH wild-type primary glioblastoma of the right frontal lobe (**B**), and a grade 4, IDH wild-type primary glioblastoma of the premotor area of the right frontal lobe. The LGG (**A**) shows no increased signal on the rCBV map, while the HGGs (**B**,**C**) present inhomogenously increased signal on the rCBV maps in the non-necrotic peripheral tumoral areas (**B**,**C**). *DSC: dynamic susceptibility contrast; PWI: perfusion WI; rCBV: relative cerebral blood volume; LGG (low-grade glioma)*.

Currently, DSC is the most widely available and performed sequence for evaluating glioma perfusion and is characterised by a short acquisition time, as compared to DCE and ASL. On the other hand, limitations of PWI-DSC are represented by T1-relaxation effects, pronounced susceptibility artefacts, and limited sensitivity for small cortical-surface lesions [28]. In contrast to ASL, which does not require gadolinium injection, DSC requires contrast administration and is based on the susceptibility-induced signal loss on T2WI caused by gadolinium reaching cerebral tissue. Subsequently, a curve framing the signal intensity over time is generated, and perfusion metrics are obtained and presented through perfusion maps. The key parameter in oncology is the relative cerebral blood volume (rCBV), which refers to the volume of blood in a given amount of brain tissue (millilitres of blood per 100 g of brain tissue) normalised to the contralateral healthy white matter [29]. Therefore, rCBV reflects the relative increase in perfusions in tumours as compared to healthy cerebral tissue. rCBV is often used to distinguish between LGGs, which present low rCBV, reflecting the lower degree of angiogenesis in these slower-growing tumours as compared to HGG, which are characterised by high rCBV, and to identify possible HGG foci within an LGG to guide tumour biopsy (Figure 4 and Figure 6) [30,31,32]. Interestingly, oligodendrogliomas grade II show mild to moderate increased perfusion, which helps the neuroradiologist in the differential diagnosis with low-grade astrocytoma, in contrast to oligodendrogliomas grade III, which demonstrate a high increase in perfusion (Figure 3G) [33]. Moreover, it is key to distinguish between vasogenic oedema and infiltrative oedema since the latter presents increased rCBV [34,35,36,37]. Finally, rCBV is an earlier biomarker to distinguish LGG progression to HGG compared to contrast-enhanced MRI [34,35,36].

#### Dynamic Contrast Enhancement

DCE is a well-known perfusion technique based on a dynamic MRI acquisition to evaluate T1 shortening induced by contrast passing through brain tissue. Main DCE limitations are based on the extended acquisition time and the absence of universal consensus on acquisition, modelling and arterial-input-function methodology [38]. Particularly, DCE quantifies the intravascular gadolinium (true perfusion) diffusion to the interstitial compartment (vascular permeability) caused by abnormal and atypical tumour neovascularity [10,39]. The most used derived parameter is the volume transfer constant (Ktrans), reflecting the capillary permeability, which has a paramount role in grading gliomas. Ktrans is increased in HGG due to their disordered neovascularity and increased capillary permeability. Moreover, ve represents fractional extracellular vascular space, which is reduced in HGGs, as compared to LGGs, due to their high cellularity [27,40].

#### Arterial Spin Labelling

ASL is an emerging perfusion technique for evaluating brain tumours, and it is being continuously investigated and implemented in clinical practice since it is based on blood water as an endogenous tracer and does not require contrast administration, which is ideal for patients suffering from renal insufficiency, allergic to gadolinium or requiring repeated follow-ups [28]. This is particularly advantageous for iLGGs, in which repeated imaging surveillance is often employed, raising concerns about the long-term effects of gadolinium exposure [39]. Moreover, ASL is immune to susceptibility artifacts and does not require complex post-processing, in contrast to DSC [28,41]. Specifically, ASL is based on the subtraction of a labelled image and a control image of the same cerebral area. Specifically, water molecules of inflowing blood proximal to the imaging slab are magnetically tagged using a radiofrequency inversion pulse that saturates them. Subsequently, labelled and control images are subtracted to eliminate the static signals, and the remaining image reflects the perfusion signals, proportional to the cerebral blood flow (CBF). Particularly, the International Society for Magnetic Resonance in Medicine (ISMRM) 2023 consensus recommendations identified the 3D Pseudo Continuous ASL (3D pcASL) with background suppression as the clinical standard [42]. As compared to pulsed and continuous ASL, pcASL offers a higher signal-to-noise ratio within feasible scan times, robustness across pathologies, and quantitative CBF maps that can be interpreted visually without demanding post-processing [28,42]. Multiple studies show that 3D pcASL-derived TBF (tumour blood flow) is able to distinguish LGGs from HGGs on the degree of microvascular proliferation, which enhances signal and is commonly increased in HGGs [34,35,36,43,44,45]. Specifically, nTBF (normalised TBF) has been proven to be the most robust quantitative index, although no clear thresholds have been identified [36].

#### 2.2.3. Magnetic Resonance Spectroscopy

Magnetic Resonance Spectroscopy (MRS) allows the in vivo measurement of brain neurometabolite levels in a specific volume of interest (VOI) of the brain, in the case of single-voxel (SV) MRS, or multiple areas of interest in the case of multi-voxel (MV) MRS. VOI should be correctly placed on the lesion by avoiding necrotic or liquoral areas or on the peripheral oedema to identify possible infiltrative oedema as compared to vasogenic oedema [10,14,46].

#### Proton (1H) Magnetic Resonance Spectroscopy

**Figure 7:**
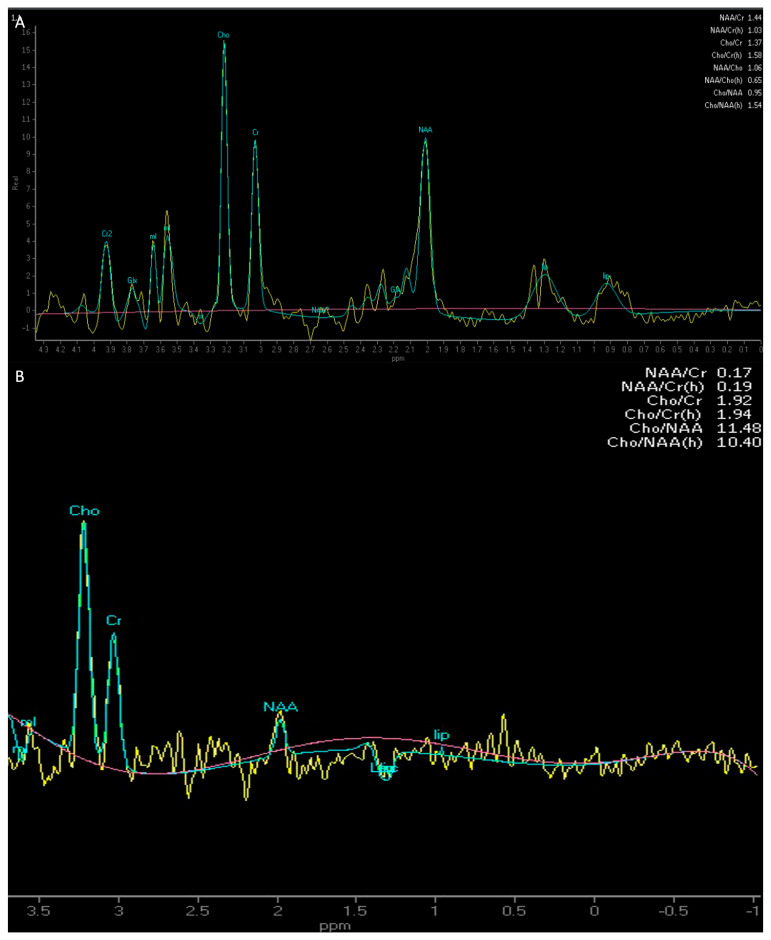
Compares the multi-voxel MRS with intermediate TE of an incidental grade 2, IDH-mutant diffuse astrocytoma involving the fronto-opercular portion of the right frontal lobe, which demonstrates a reduction in NAA and an increase in Cho peaks with inversion of the Cho/NAA ratio (**A**), and a grade 4, IDH wild-type primary glioblastoma of the right frontal lobe, which demonstrates a very high peak of Cho and a decreased NAA peak, resulting in the inversion of the Cho/NAA ratio (**B**). *Cho: choline; NAA: N-Acetyl-Aspartate; MRS: magnetic resonance spectroscopy; TE: echo time*.

Proton (1H) MRS is the most commonly performed technique, and although it can be performed with long (288 ms or 144 ms) or short (35 ms) echo times, the long echo time is the most widely performed since it better depicts a larger number of neurometabolites [10,14]. On the spectrum, neurochemicals are represented by typical peaks at specific frequencies used for neurometabolite identification [46]. LGGs commonly present a slight reduction in NAA (N-acetylaspartate), which is a marker of neuronal integrity and mitochondrial function, and a modest increase in Cho (Choline), which is a marker of membrane synthesis and cell turnover. Sometimes, LGGs present increased mI (myo-inositol), which is a glial marker and osmolite, and the mI/Cr (creatine) ratio. Cr gives indications on cellular energetic metabolism and is often used as a reference for other metabolites [8,47]. On the other hand, HGGs present a significant decrease in NAA and an increase in Choline, together with decreased mI and Cr. The identification of specific areas of markedly increased NAA and decreased Cho in LGG may suggest foci of high-grade tumour and effectively guide lesion biopsy [48]. Increased Cho/Cr and decreased NAA/Cho ratios suggest HGGs. The increase in the normally absent neurometabolites in LGGs, such as lactate, which reflects anaerobic glycolysis, and lipids, which reflect increased membrane synthesis and cell turnover, is typical of HGGs. Therefore, the novel identification of these peaks on the spectrum of LGGs suggests tumour progression (Figure 7) [10,49].

#### 2-Hydroxyglutarate Magnetic Resonance Spectroscopy

From a metabolic perspective, isocitrate dehydrogenase (IDH) converts isocitrate to α-ketoglutarate (αKG) in the cytoplasm (IDH1) and the mitochondria (IDH2). IDH1/2-mutated gliomas generate 2-hydroxyglutarate (2HG) and induce DNA hypermethylation. Therefore, 2HG is highly concentrated in IDH-mutant gliomas, most commonly LGGs. Since 2HG presents a small peak, which is difficult to identify between GABA and glutamate/glutamine peaks, it requires hyperpolarised 3T MR scanners and long post-processing, which limit the widespread use of the technique [48,50,51,52,53].

#### 2.2.4. Susceptibility-Weighted Imaging

**Figure 8:**
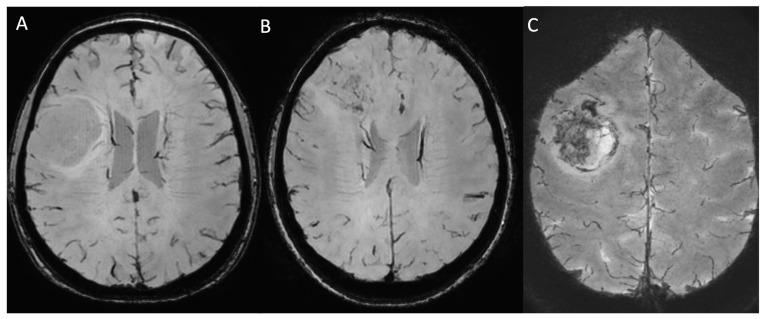
Compares the SWI sequences of patients affected by a grade 2, IDH-mutant diffuse astrocytoma involving the fronto-opercular portion of the right frontal lobe (**A**), a grade 4, IDH wild-type primary glioblastoma of the right frontal lobe (**B**), and a grade 4, IDH wild-type primary glioblastoma of the premotor area of the right frontal lobe (**C**). No “blooming” foci are present in the SWI sequence of the LGG (**A**), while HGGs show inhomogeneously decreased signal intensity in the tumoral vital areas, suggestive of neovascularisation. *SWI: susceptibility weighted image; LGG: low-grade glioma; HGG: high-grade glioma*.

Susceptibility-weighted imaging (SWI) is an MRI technique showing high sensitivity to differences in magnetic susceptibility between tissues. Therefore, substances that cause distortions of the magnetic field, such as calcium, iron, deoxyhemoglobin and meta-haemoglobin, appear hypointense. In particular, the combination of magnitude data and filtered phase mapping allows the distinction between haemorrhage and calcium. Particularly, paramagnetic substances, such as iron and haemorrhage, appear hypointense on the phase map in contrast to diamagnetic substances, such as calcifications, which appear hyperintense on the filtered phase map [54]. The identification of calcifications is useful in the differential diagnosis of iLGGs since dense and coarse calcifications are prevalent in oligodendrogliomas and rare in astrocytomas (Figure 3F) [8]. On the other hand, SWI and the related minimum intensity projections (MIPs) allow the study of internal vascular architecture and intratumoral haemorrhage. These findings are key in differentiating LGGs from HGGs since increased neovascularity and haemorrhagic foci suggest HGGs, especially in the case of necrotic foci. Similarly, areas of neovascularity may indicate a higher-grade component within an LGG and guide biopsy (Figure 8) [19,55]. The Intratumoral Susceptibility Signals (ITSS) is a score derived from SWI, which quantifies the degree of intratumoral susceptibility based on the number of linear or dot-like structures in the maximum cross-section of tumours on SWI (Grade 0: no ITSS; Grade 1: 1–5 dot-like or fine linear ITSS; Grade 2: 6–10 dot-like or fine linear ITSS; and Grade 3: 11 dot-like or fine linear ITSS) [56]. Recent literature demonstrated a correlation between the ITSS score and glioma grade. Particularly, HGGs showed higher ITSS scores as compared to LGGs [56], suggesting that SWI may contribute to glioma grading [57]. Moreover, Park et al. demonstrated that the degree of ITSS shows a significant correlation with the value of PWI-derived rCBV, and its diagnostic performance for glioma grading was comparable with that of DSC [57]. Finally, Grabner et al. introduced the SWI-based LIV (Local Image Variance) score to quantify hypointensities. LIV is a measure of image variation proximal to a pixel, which increases in cases of a high density of blood vessels or microbleeds represented as signal loss on SWI images. The authors identified a correlation between SWI LIV and glioma grading; namely, high LIV was correlated to HGGs [58].

#### 2.2.5. Diffusion-Weighted Imaging and Apparent Diffusion Coefficient Map

**Figure 9:**
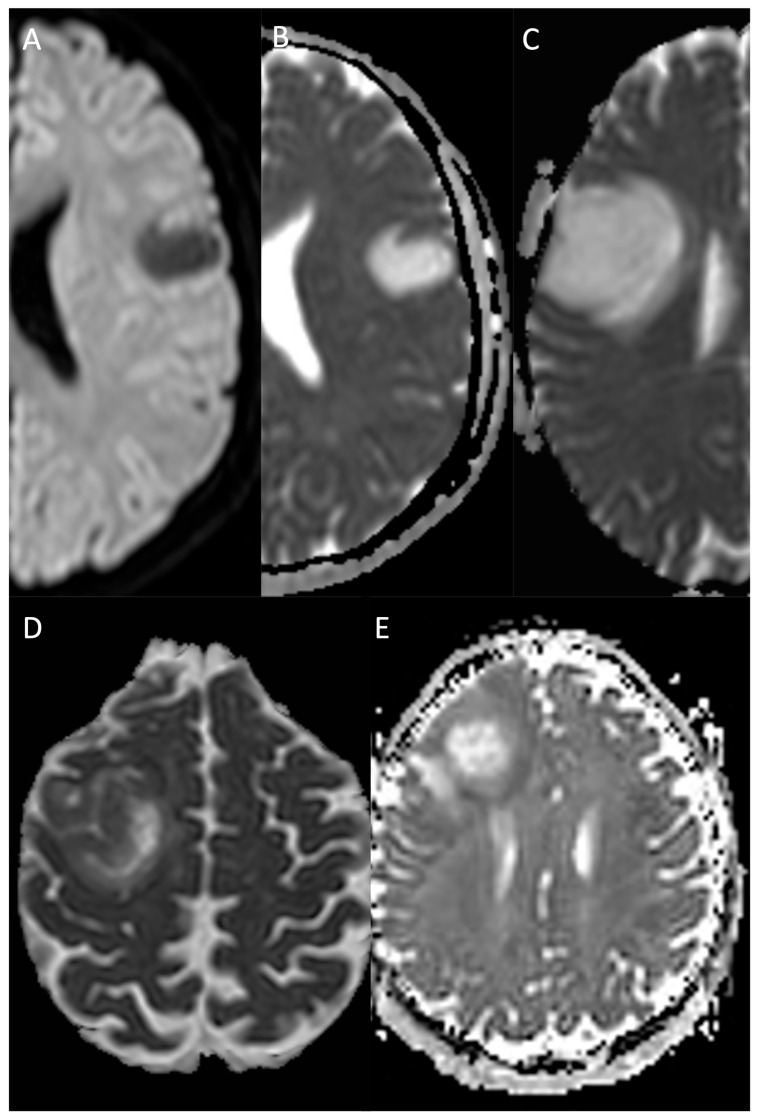
Compares the DWI/ADC sequences of patients affected by an incidental grade 2, IDH-mutant diffuse astrocytoma involving the inferior and lateral (fronto-opercular) portion of the left frontal lobe (**A**,**B**), a grade 2, IDH-mutant diffuse astrocytoma involving the fronto-opercular portion of the right frontal lobe (**C**), a grade 4, IDH wild-type primary glioblastoma of the premotor area of the right frontal lobe (**D**), and a grade 4, IDH wild-type primary glioblastoma of the right frontal lobe (**E**). LGGs (**A**–**C**) do not show diffusion restriction, while HGGs (**D**,**E**) demonstrate significant diffusion restriction of the peripheral vital areas. *DWI: diffusion-weighted image; ADC: apparent diffusion coefficient; LGG: low-grade glioma; HGG: high-grade glioma*.

Diffusion-weighted imaging (DWI) is an advanced MRI technique that reflects the Brownian random motion of water molecules within tissues at a microscopic level. In healthy conditions, the diffusion is “isotropic”, namely, there are no areas of restricted diffusion, which would define the “anisotropic diffusion” [10]. Restricted water diffusion appears hyperintense on DWI sequences and hypointense on the corresponding Apparent Diffusion Coefficient (ADC), offering valuable information regarding iLGGs’ cellularity, heterogeneity, peritumoral oedema, white matter integrity and post-treatment injuries [59]. Particularly, low ADC values have been identified in core lesions and peripheral oedema of HGGs as compared to LGGs, suggesting a potential role for DWI/ADC in grading these tumours and identifying the peripheral oedema’s infiltrative nature [59,60]. Moreover, low ADC areas in a heterogeneous lesion may indicate a high-grade focus and direct tissue sampling [61]. Notably, cystic areas, which are more common in LGGs, and necrotic areas, which are prevalent in HGGs, present high ADC values, corresponding to areas of increased free water diffusion (Figure 9) [8].

#### 2.2.6. Diffusion Tensor Imaging and Tractography

**Figure 10:**
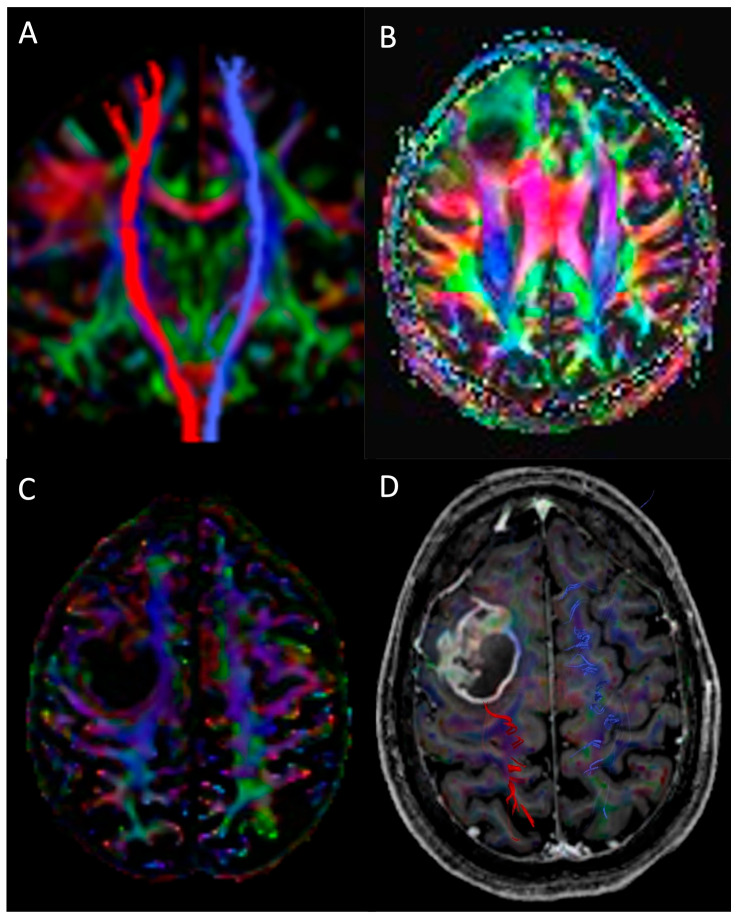
Shows the DTI-derived FA map of a grade 2, IDH-mutant diffuse astrocytoma involving the fronto-opercular portion of the right frontal lobe, which demonstrates no disruption of the right cortico-spinal tract (**A**), and the DTI-derived FA maps of a grade 4, IDH wild-type primary glioblastoma of the right frontal lobe, which demonstrates the disruption of the white matter tracts involved and surrounding the HGG (**B**). (**C**) Shows the DTI-derived FA map of a grade 4, IDH wild-type primary glioblastoma of the premotor areas of the right frontal lobe, which shows disrupted white matter tracts caused by the lesion, which is anterior to the right cortico-spinal tract. These findings are easily detectable when the DTI-derived FA map is superimposed on the post-contrast T1WI (**D**). *DTI: diffusion tensor imaging; FA: fractional anisotropy; WI: weighted image; HGG: high-grade glioma*.

DTI (Diffusion Tensor Imaging) is based on the evaluation of the three-dimensional brain diffusion based on the parameters direction (eigenvectors) and diffusivity (eigenvalues). The diffusion along white matter tracts (WMTs) is anisotropic; namely, the highest diffusivity is parallel and the lowest is perpendicular to the direction of the fibre bundles in healthy conditions of axonal myelination and axonal membrane integrity and in preserved white matter tracts [10,62]. The key DTI parameters are FA (fractional anisotropy), measuring the directionality of diffusion, ranging from anisotropic (1) to isotropic (0), and MD (mean diffusivity), measuring the magnitude of diffusion. Another important parameter is the NS (number of streamlines), reflecting the axonal density in the fibre bundle [8,12]. Tractography consists of the 3D visual representation of WMTs in the brain through a colour-coded map. Particularly, antero-posterior fibres are shown in green, cranio-caudal fibres are shown in blue and transverse fibres, between the hemispheres, in red [14]. DTI and tractography have been frequently applied to pre-surgical tissue characterisation and grading, pre-surgical planning and prediction of post-surgical neurological deficits, and intraoperative evaluation of glioma location and resection [63,64,65,66,67]. HGGs tend to cause a higher decrease in FA and NS and an increase in MD in the lesion core and in the peripheral infiltrative oedema as compared to LGGs. That is explained by the intrinsic nature of HGGs and LGGs. HGGs tend to disrupt WM fibres and interrupt the bundles, while LGGs tend to compress or dislocate fibre bundles, preserving WM integrity (Figure 10) [12,68]. Indeed, tractography proved to be capable of effectively delineating the course and integrity of WM tracts by distinguishing between WM disruption and displacement to offer an optimal prediction of the spatial relationship between the lesion and the neural networks to the surgeon [10,68,69,70]. Moreover, tractography is paramount to delineate tumour boundaries and relations with eloquent areas, such as language, vision or motor control, leading to greater and safer resection with improved long-term outcomes [71,72]. For iLGGS adjacent to language-eloquent brain areas, Zoli et al. demonstrate that performing tractography of the arcuate fasciculus is particularly valuable in guiding surgical planning and ensuring the preservation of language capabilities [73]. Interestingly, Campanella et al. demonstrated that specific alterations in white matter tracts, as identified by tractography, could discriminate between tumour histotypes, thus supporting the diagnostic potential of DTI in characterising glioma subtypes [74]. Finally, D’Andrea et al. also demonstrated that intra-operative DTI is a safe and effective alternative approach to awake surgery, and Vassal et al. suggested that the combination of intra-operative DTI and subcortical direct electrical stimulation grants an optimal resection of tumours located in eloquent motor areas, with low morbidity for patients [75,76]. These data are paramount to provide an optimal surgical plan with the goals of achieving radicality and preserving neurological functions to provide the patients with a high quality of life [75].

#### 2.2.7. Functional MRI

**Figure 11:**
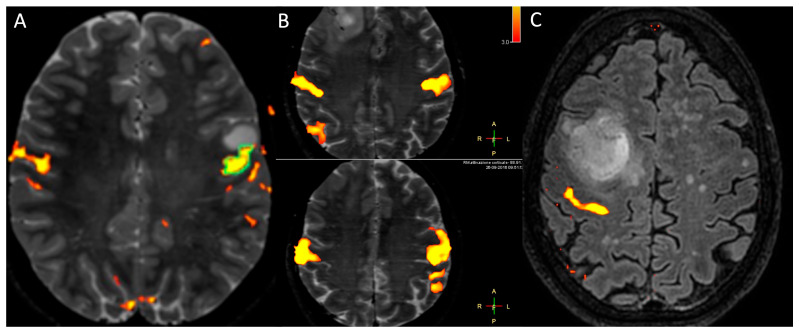
Compares the fMRIs of: an incidental grade 2, IDH-mutant diffuse astrocytoma involving the inferior and lateral (fronto-opercular) portion of the left frontal lobe, whose task-based fMRI study shows a cluster of mouth activation close to the posterior margin of the lesion (**A**); a grade 4, IDH wild-type primary glioblastoma of the right frontal lobe, task-based fMRI study shows a bilateral cluster of mouth activation (**B**); and a grade 4, IDH wild-type primary glioblastoma of the premotor area of the right frontal lobe, task-based fMRI study shows a cluster of activation corresponding to the left hand, whose brain area appears close but not infiltrated by the lesion (**C**). *fMRI (functional MRI)*.

Functional MRI (fMRI) is an advanced MRI sequence that indirectly measures the neuronal activity in relation to brain regional differences in blood flow, thanks to the blood oxygenation level dependent (BOLD) imaging. Particularly, BOLD is based on the magnetic properties of deoxygenated blood, which is paramagnetic, and oxygenated blood, which is diamagnetic. When a specific brain area is activated, it consumes a significant amount of oxygen, causing an increase in deoxygenated haemoglobin and a decrease in oxygenated haemoglobin. To cope with the increased oxygen demand, there is a subsequent increase in blood flow (CBF) to the brain area, resulting in an increase in oxygenated haemoglobin and a decrease in deoxygenated haemoglobin. This phenomenon generates an increase in the BOLD signal, indicating the activation of that specific brain area [8,10]. fMRI may be task-based, namely, patients are asked to perform a specific task during MRI acquisition, or a resting-state fMRI, namely, patients do not perform any tasks. Although there are promising studies that demonstrate the reliability of rs-fMRI in mapping the sensorimotor network and suggest that rs-fMRI may be a useful tool for surgical pre-planning, its role is mainly related to research purposes [77,78,79]. In the oncological field, task-based fMRI is the most widely performed [80,81] and has been performed both for investigating glioma grading and for pre-surgical planning. Decreased BOLD signal and a shorter time-to-peak (TTP) have been identified in the cerebral cortex infiltrated by HGGs, as compared to LGGs [82]. Similarly, Hou et al. demonstrated a decreased fMRI activation in tissues adjacent to HGG and hypothesised that the neovascularity of the tumour may cause the decoupling between CBF and neuronal activity [83]. Therefore, fMRI is not considered a biomarker for grading gliomas, and its main role encompasses the pre-surgical planning. Particularly, patients undergo task-based fMRI to localise eloquent areas to obtain language, vision, memory and sensory-motor mapping, which retain significant implications in intraoperative navigation and surgery, as well as in patients’ prognosis and quality of life [13,81,84,85]. In fact, incorporating fMRI in the pre-surgical planning and integrating fMRI data with neuronavigation systems have been shown to improve long-term survival rates in patients with iLGGs situated in eloquent brain regions [86] because fMRI helps surgeons in maximising the extent of tumour resection and minimising the causing of new or worsening patients’ neurological deficits (Figure 11) [87,88]. Finally, fMRI may guide the delineation of a seed region for tractography reconstruction [89,90].

## 3. The Role of AI in Glioma Detection, Grading and Prediction of Genetic Profile

Artificial intelligence (AI) has been increasingly implemented in medicine and healthcare. Radiology and neuroradiology are privileged medical branches for AI applications, as they encompass the production of a massive quantity of data in the form of medical images. Among the applications of AI, and for the purpose of our review, we will focus on the differential diagnosis of HGGs and LGGs.

### 3.1. Machine Learning and Deep Learning

Machine learning (ML), a rapidly evolving type of AI, holds the paramount potential to impact the early and differential diagnosis of iLGGs. ML algorithms acquire knowledge by analysing images and extracting relevant features. ML includes two learning procedures: unsupervised learning, in which algorithms identify patterns in unlabelled data, and supervised learning, in which algorithms learn to map labelled inputs, such as segmented images [91,92]. Deep learning (DL) is a subtype of ML, which is frequently applied to neuroradiology to support various tasks, from glioma differential diagnosis to treatment decisions. DL models are commonly based on artificial neural networks (ANNs) and characterised by multiple layers of interconnected “artificial neurons”, namely computational units that learn to extract specific features in a hierarchical modality. Particularly, these layers are organised in the following modality: the lower-level layers learn basic image features, such as edges and textures, and subsequent higher-level layers combine these features to represent more complex anatomical structures or pathological patterns. The final layer is called the output layer and offers the required result, such as glioma classification or grading [93,94].

### 3.2. Radiomics and Radiogenomics

Radiomics is a research field that is based on the extraction of a large number of features from images using data characterisation algorithms. These radiomic features may be of many kinds, from morphological to functional features, and need to be carefully evaluated to select the key radiomic features to be analysed [91,95]. The typical pipeline for radiomic studies encompasses: (1) image acquisition and pre-processing, such as artifact correction, (2) region of interest (ROI) segmentation, (3) feature extraction, (4) key feature selection based on statistical analyses and/or ML/DL algorithms, (5) predictive model development, namely the process of training a model with the selected features to predict the outcome of interest, (6) model validation, namely the process which assesses model performance through internal validation, such as cross-validation, or external validation, using an independent dataset, (7) integration of the model into clinical practice, namely the model application in daily routine for glioma grading and differential diagnosis [17,91].

On the other hand, radiogenomics is a translational field which correlates features extracted from radiological images to genetic data, such as gene expression or mutations. While radiomics mainly focuses on extracting a large number of features from medical images to develop and validate a prediction model to be used in clinical practice, radiogenomics enhances and improves this procedure by investigating how these radiomic features can be related to glioma genetic information. This integration of imaging and genetics provides a more comprehensive understanding of gliomas and could be the key to unravelling tumour genetics, identifying imaging biomarkers, and supporting personalised medicine [96,97,98].

Multiple studies demonstrated that AI-based algorithms applied to standard and advanced MRI sequences are valuable tools in the differential diagnosis of iLGGs [99,100]. Particularly, radiomics texture analysis has proved to be extremely efficient in differentiating LGGs from HGGs [101,102,103], and DL algorithms have proved their efficacy in identifying peritumoral white matter infiltration [104]. Moreover, recent papers used radiomics algorithms to distinguish tumours in relation to their genetic profiles, and optimal results have been obtained [18,98]. Habould et al. produced an automated segmentation-based radiomics algorithm, which also incorporated clinical and laboratory data, to differentiate HGGs from LGGs and to predict the glioma molecular status in relation to IDH, ATRX and MGMT mutation and to 1p19q codeletion [16]. Conversely, Shnoul et al. focus their analysis on LGGs and investigate the IDH, ATRX, and TERT mutations, MGMT methylation status and 1p19q codeletion with sufficient accuracy [105]. Also, radiogenomic studies investigated the genetic signature of gliomas. Particularly, Li et al. combined genomic data with radiomic-based glioma texture analysis extracted from T2WI to predict EGFR levels in LGGs with an AUC of 0.95 [106]. Interestingly, Gao et al. demonstrated the importance of the human–AI synergy by showing that the combined accuracy of the neuroradiologist and DL was superior to the separated accuracies in tumour differentiation and grading [107]. Future studies are needed, yet the premises are promising.

### 3.3. Limitations

The implementation of AI, radiomics and radiogenomics in daily routine glioma management is significantly hampered by technical, methodological, and systemic limitations. These limitations and challenges should be addressed to enhance AI reliability and applicability to radiology and medical practice.

#### 3.3.1. Standardisation Challenges in Image Acquisition

Worldwide, there is significant variability in brain MRI acquisitions related to different scanners and protocols, causing inconsistencies in data quality and quantity, depending on MRI image resolution and contrast, slice thickness, patient positioning, etc. [108]. This variability impacts the reproducibility of radiomic features and the generalizability and reliability of models across diverse institutions and represents a challenge for multicentric studies. While techniques like re-sampling to a common voxel resolution and intensity normalisation are employed to mitigate these issues, standardised and reproducible guidelines are necessary for the goal of standardisation and generalizability of AI models [108,109].

#### 3.3.2. Segmentation and Feature Extraction Variability

Challenges in tumour segmentation and the robustness of feature extraction also present significant hurdles. Accurate delineation of ROIs (regions of interest) and VOIs (volumes of interest) is a critical prerequisite for radiomic analysis because if the initial segmentation is inaccurate due to manual segmentation, inter-rater variability, or biased automated methods, the extracted features are unreliable, directly affecting the predictive power of downstream radiomics models [110]. Furthermore, ensuring fully automated, non-redundant feature extraction remains a challenge, especially for deep features derived from complex neural networks, which demand exceptionally large sample sizes for reliable model development [111].

#### 3.3.3. Data Scarcity and Single-Omics Analysis

AI algorithms are trained on big data and suffer from data scarcity and heterogeneity. Obtaining large, diverse, and well-annotated datasets is challenging, particularly for multi-omics analysis, such as radiogenomics, which requires both imaging and comprehensive genomic data [109,112]. 

Currently, most research involves single-omics analysis (e.g., solely radiomics or genomics), which has inherent limitations. This approach may not fully capture the complex clinical and biological characteristics of glioma, which are influenced by pathology, genes, medical imaging, and clinical aspects. A comprehensive diagnosis and accurate grading, especially for complex and heterogeneous tumours like gliomas, require a multi-dimensional understanding that single-omics analysis cannot fully provide, thereby limiting the predictive power and clinical utility of such models [113].

#### 3.3.4. Model Reproducibility and Generalizability

Deficiencies in model reproducibility, generalizability, and validation represent a major challenge to clinical implementation [114]. Reproducible and generalisable models across diverse patient populations and imaging protocols are extremely complex to offer since most models are trained and validated on single-centre datasets, which fail to account for inter-centre variability, leading to overfitting and poor performance when applied to external data [113]. Moreover, publicly available datasets may suffer from quality issues, including images from older protocols or potential segmentation errors, further compromising model accuracy and reliability in real-world settings [115]. 

#### 3.3.5. Black Box and Interpretability Concerns

The black box nature of many AI and deep learning models used in radiomics consists of their internal decision-making processes, which are opaque and not entirely intelligible for neuroradiologists [114]. This lack of interpretability represents a barrier to clinical adoption, as radiologists require transparency and trust in diagnostic and prognostic tools in order to offer optimal management of patients’ gliomas [113]. Moreover, these AI tools raise ethical and legal concerns about accountability in the case of misdiagnoses related to non-intelligible AI models [116]. Clinicians require clear, comprehensible explanations for AI-generated insights to build trust and integrate these tools responsibly into patient care.

#### 3.3.6. Ethical and Privacy Issues

The increasing integration of AI, radiomics, and radiogenomics into healthcare, and especially in neuroradiological contexts such as glioma diagnosis and characterisation, introduces significant ethical and privacy challenges that demand careful consideration. Medical images, clinical records, and genomic data used for AI model training contain highly sensitive personal health information. The aggregation of such vast and detailed datasets, even when de-identified, raises substantial risks of re-identification due to advanced data mining technologies and the high dimensionality of the combined information. This issue requires the identification of guidelines, which standardise the de-identification process and limit identifiers, and explore privacy-preserving techniques like differential privacy, consisting of the obscuration of patient-level information while preserving population-level regularities essential for model training [113]. Moreover, informed consent for data collection and its subsequent secondary use is a hot topic in this field. Specifically, prospective data collection from patients requires informed consent, while the common practice of retrospectively reusing de-identified clinical data for AI model development is still an ethically related issue. Automated and clear mechanisms for notifying subjects about their data’s potential inclusion in AI datasets and providing opportunities for them to withdraw data appear to be a viable solution [115]. 

Finally, ethical implications and fairness in AI models are paramount to ensure equitable healthcare delivery. If AI training datasets are not sufficiently representative of the diverse patient populations, such as lacking data from certain demographic groups or with specific socioeconomic backgrounds, the AI models can exacerbate existing health inequalities [117]. This can lead to biased predictions, suboptimal care, or misdiagnosis for marginalised populations. Therefore, fairness with transparent communication of model limitations and performance across different demographic subgroups is crucial for the ethical and equitable clinical implementation of AI, radiomics, and radiogenomics in glioma management [116]. 

### 3.4. Future Directions

The trajectory of AI, radiomics, and radiogenomics in glioma management points towards overcoming current limitations through advanced data integration, collaborative frameworks, and enhanced model transparency, ultimately aiming for truly personalised and effective patient care, with a strong focus on improving glioma grading and differential diagnosis.

#### 3.4.1. Enhanced Standardisation and Multi-Institutional Validation

Enhanced standardisation and multi-institutional validation efforts, addressing the current lack of standardisation in image acquisition, processing, and feature extraction, are essential for AI integration into clinical practice [118]. Future efforts will focus on adopting optimised standard imaging processes, establishing common criteria for segmentation, and ensuring robust, non-redundant feature extraction. Multi-centre studies involving heterogeneous populations are mostly needed to enhance model validation and generalizability [119]. This will enable the creation of robust, clinically trustworthy tools that can be deployed widely across different healthcare systems, accelerating the pace of innovation and clinical impact. Finally, international guidelines are required to provide frameworks for consistency and reproducibility [118]. 

#### 3.4.2. Advancements in Multi-Omics Integration

A significant future direction involves advancements in multi-omics data integration, namely the comprehensive fusion of multi-omics data, such as genomics, transcriptomics, proteomics, and pathomics, with radiomics, for a comprehensive understanding of glioma [113]. By capturing the full biological complexity of the tumour, multi-omics integration promises to provide a more complete picture of the pathology to enable a more accurate prediction of genetic mutations, prognosis, and treatment response in glioma patients [120]. This approach can help identify specific therapeutic targets and predict drug resistance towards personalised, holistic medicine [121].

#### 3.4.3. Explainable AI and Federated Learning

Explainable AI (XAI) is the current ambitious challenge to address the “black box” issue and facilitate AI integration into clinical practice. XAI aims to make AI models more transparent and interpretable, providing comprehensible explanations for their predictions and building trust among clinicians [114,122]. Federated learning (FL), which is central to fostering collaborative research and AI clinical implementation, is emerging as a potential solution to data scarcity and privacy concerns [123]. FL enables collaborative model training across multiple institutions without centralising sensitive patient data and allows for the development of robust, generalizable models from diverse, distributed datasets [123]. This synergistic combination of XAI and FL is critical for scaling AI solutions in healthcare while maintaining ethical standards and clinical utility [124].

#### 3.4.4. Integration into Clinical Workflow and Decision Support

The ultimate goal is the integration of AI models into clinical workflow and decision support systems. This involves seamlessly integrating AI, radiomics, and radiogenomics into routine clinical practice as decision-support tools and needs user-friendly interfaces and systems that can provide clinicians with quantitative, objective information to augment their subjective assessments and guide clinical decision making [125]. This shift implies a move from qualitative, subjective assessments to quantitative, objective, and data-driven insights for every patient, enhancing the accuracy and consistency of diagnosis, characterisation, and grading [125]. Such integration could improve longitudinal diagnosis during treatment, predict tumour recurrence, and help both patients and providers set realistic expectations regarding prognosis. Finally, AI is meant to support human expertise, facilitating more informed and efficient patient management. 

## 4. The Role of MRI, Radiomics and Radiogenomics in Unravelling Glioma Genetic Profile

The 2021 WHO classification of brain tumours represents a significant revolution in the oncological field by emphasising the role of molecular, genetic and pathogenetic advances in classifying brain tumours [7]. Therefore, advanced MRI sequences have been deeply investigated to identify features or parameters that may correlate with brain tumour genetic profiles [8,9]. Although glioma genetic profiles are still the subject of ongoing research, we tried to summarise existing knowledge in the field for the various specific genetic alterations (Table 2).

### 4.1. IDH

IDH (Isocitrate Dehydrogenase) is a molecule whose key role is regulating HIF-1α (Hypoxia-Inducible Factor 1-Alpha) and therefore angiogenesis. Mutation of IDH downregulates HIF-1α and neoangiogenesis, which is crucial in tumoural growth [126]. Accordingly, IDH-mutant gliomas have been correlated with lower rCBV measured by PWI-DSC and lower Ktrans, vp (plasma volume), and ve measured by PWI-DCE, as compared to IDH wild-type gliomas [8,127,128,129,130,131]. Moreover, IDH-wild-type gliomas demonstrated a higher TBF/nTBG measured by the ASL as compared to IDH-mutant gliomas [132,133,134]. Moreover, hyperpolarized MRS may guide the identification of IDH-mutant gliomas, most commonly LGGs, thanks to the identification of 2HG, a neurochemical which is part of IDH metabolism [53]. Novel approaches to distinguish IDH-wild-type and IDH-mutant gliomas are based on CEST (Chemical Exchange Saturation Transfer) imaging. CEST MRI contrasts consist of the spontaneous chemical exchange between protons of free water and solute-bound protons. This exchange mostly depends on the concentration of endogenous proteins in the cells. Moreover, CEST signals reflect tissue microenvironment, such as pH, which affects proton exchange properties [135]. This approach is particularly interesting since it avoids the injection of gadolinium and is safe in patients presenting contraindications to contrast agents. APT (Amide Proton Transfer) is the most common type of chemical exchange saturation transfer technique [136]. Jian et al. demonstrated that IDH1-wild-type gliomas were associated with higher APTw signal intensity as compared with IDH1-mutant gliomas at 3T [137], which was confirmed by Paech et al., who obtained similar results using APT CEST MRI at 7T [135]. SWI has been proposed as a valuable tool to differentiate LGGs from HGGs. Kong et al. demonstrated that the SWI-derived ITSS score of IDH1-mutant gliomas was lower than wild-type gliomas, without any correlation with 1p19q codeletion [56]. On the other hand, Grabner et al. demonstrated that the SWI-derived LIV was increased in IDH-wild-type gliomas [58]. Some studies tried to correlate the ADC values to genetic alterations in gliomas and identified higher ADC values in IDH-mutant gliomas as compared to IDH-wild-type gliomas [98,138]. Also, standard MRI sequences may support the identification of IDH mutation. In fact, IDH-mutant, 1p19q non-codeleted astrocytomas present a highly specific sign, namely the “T2-FLAIR mismatch” characterised by global T2WI lesion hyperintensity paired with relatively FLAIR core hypodensity and peripheral hyperintensity (Figure 1). Unfortunately, this sign demonstrated low sensitivity [21]. Several AI-based models, predominantly radiomics and DL algorithms, efficiently demonstrated outstanding potential in differentiating IDH mutant from IDH wild-type gliomas [15,16,105,139]. Particularly, Chang et al. demonstrated the IDH1 mutation status with a 94% accuracy and correlated it with specific imaging features such as tumour margins, T1 and FLAIR suppression, presence of necrosis and textural features, and extent of oedema [140].

### 4.2. EGFR

EGFR (Epidermal Growth Factor Receptor) amplification favours tumour neoangiogenesis and accelerates proangiogenetic growth factors. EGFR signalling cascade is intercalated in the regulation of cell proliferation, differentiation and cancer development [141,142]. Brain gliomas characterised by EGFR amplification usually show increased rCBV originating from PWI-DSC and increased Ktrans and vp originating from PWI-DCE [127,143,144]. Qiao et al. demonstrated that EGFR-amplified gliomas show an increased ASL-CBF as compared to non-EGFR-amplified gliomas [145]. Also, DWI/ADC may support the identification of EGFR amplification since EGFR-amplified gliomas show lower mean ADC values as compared to EGFR-wild-type gliomas. These data are understandable since EGFR amplification induces cellular growth and proliferation, and ADC reflects the related increased glioma cellularity [141,146]. AI applications to MRI sequences with the scope of identifying EGFR mutations have been increasing recently. In particular, a radiogenomic study based on the evaluation of glioma texture analysis extracted from T2WI succeeded in predicting EGFR levels in LGGs with an AUC of 0,95 [106].

### 4.3. TERT

The TERT (Telomerase Reverse Transcriptase) promoter is located on the short arm of chromosome 5 and encodes for the hTERT component of the enzyme telomerase, which lengthens and maintains telomeres. Mutations of the TERT promoter have been identified in multiple tumours since they enhance the activity of telomerase and lengthen telomeres, therefore altering cellular longevity and replicative potential [147]. Based on the 2021 WHO classification of brain tumours, TERT promoter mutation is one of the molecular criteria for diagnosing IDH-wild-type glioblastoma [7]. Moreover, TERT mutation has been associated with resistance to anti-growth factors and increased angiogenesis, which pair with the higher DSC-rCBV and DCE-ve in TERT promoter mutant IDH-wild-type gliomas [8,127]. TERT mutation has also been investigated with radiomics. Particularly, based on the LASSO (least absolute shrinkage and selection operator) regression model, which is an automated ML approach that automatically selects the best predictive features from the cohort, multiple radiomic features, such as the MRS-related Cho/Cr ratio and Lac, and the percentage of core necrotic volume, were significantly higher in TERT-mutated gliomas as compared to TERT wild-type gliomas [148]. Finally, radiomics models based on multiparametric MTI confirmed that AI-based tools are efficient in identifying pre-operative TERT promoter mutation status [149].

### 4.4. MGMT

MGMT (O6-Methylguanine-DNA methyltransferase) is an enzyme that removes the methyl group added to DNA by temozolomide and reduces the damage which would have led to tumour cell death [150]. Methylation of the MGMT promoter, which is common in LGG and commonly associated with IDH mutation, decreases its activity and improves the response to temozolomide and patients’ overall survival [151,152]. MGMT-methylated gliomas showed decreased DSC rCBV, whereas unmethylated brain gliomas demonstrated increased DCE Ktrans and ve [126,127,153,154]. Moreover, MGMT-methylated gliomas demonstrated a higher CBF measured by the ASL, as compared to LGGs [40]. APTw CEST MRI at 3T may be a valuable tool to assess MGMT promoter methylation status, since Jian et al. demonstrated that GBMs (Glioblastomas) with unmethylated MGMT promoters present higher APTw signal as compared to methylated GBMs [155]. As per IDH mutation, SWI represents a valuable tool in differentiating MGMT promoter mutations in gliomas. Kong et al. demonstrated that the SWI-derived ITSS score of MGMT methylated gliomas was lower than unmethylated MGMT gliomas, and there was no correlation with 1p19q codeletion [56]. The DWI/ADC sequence contributes to genetically differentiating gliomas, since gliomas presenting a methylated MGMT promoter demonstrated higher ADC values [156,157]. Together with IDH, MGMT promoter methylation is among the most investigated genetic biomarkers with radiomics. In particular, radiomics and deep learning algorithms demonstrated with good to moderate accuracy that MGMT promoter methylation may be identified thanks to radiomic features [15,16,105]. Particularly, Chang et al. demonstrated the MGMT promoter methylation status with 83% accuracy and correlated it with specific imaging features such as tumour margins, T1 and FLAIR suppression, presence of necrosis and textural features, and extent of oedema [140].

### 4.5. 1p/19q Codeletion

The 2021 WHO classification of brain tumours defined oligodendrogliomas as genetically presenting an IDH1/2 mutation and the codelection of chromosome arms 1p and 19q [7]. Li et al. described the SWITW sign on T2WI as a sensitive and specific biomarker to diagnose IDH-mutant 1p/19q codeleted oligodendrogliomas, which benefit from a conservative surgery and show a good response to chemoradiotherapy and improved outcome [22]. Recent literature identified increased DSC-CBV in IDH-mutant 1p19q codeleted oligodendrogliomas grade 3, as compared to IDH-mutant 1p19q non-codeleted gliomas in relation to the typical increased vascularisation of oligodendrogliomas and decreased DSC-CBV, as compared to IDH-wild-type GBMs (glioblastomas) [129,158,159,160,161,162]. Similarly, grade 2–3 oligodendrogliomas presented an increased DCE Ktrans and ve as compared to grade 2–3 astrocytomas and decreased DCE Ktrans and ve, as compared to GBMs [161,162]. 1p/19q codeletion has been frequently investigated through AI-based models, which demonstrated overall moderate to high accuracy depending on the tool and increased accuracy when radiologists have access to DL results [15,16,105]. Particularly, Chang et al. demonstrated the presence of 1p/19q codeletion with a 92% accuracy and correlated it with specific imaging features such as tumour margins, T1 and FLAIR suppression, presence of necrosis and textural features, and extent of oedema [140]. 

### 4.6. H3-K27M

The K27M mutation in histone H3 is a biomarker of paediatric diffuse midline gliomas, which present aggressive growth patterns and resistance to therapy [7]. Chen et al. demonstrated that H3 K27M-mutant gliomas presented significantly lower values of minimal ADC, peritumoral ADC, ratio of minimal ADC, and ratio of peritumoral ADC values as compared to wild-type gliomas and suggested that the combination of ratio of minimal ADC and ratio of peritumoral ADC can non-invasively detect the H3 K27M mutational status in brain diffuse midline gliomas [163]. Unfortunately, advanced MRI sequences are not always available, and AI is trying to bridge the gap thanks to the application of DL algorithms to standard MRI sequences in order to identify the H3-K27M mutation. Li et al. developed an automatic DL algorithm for predicting the H3-K27 mutation on T2WI, obtaining optimal accuracy (92.1%), sensitivity (98.2%) and specificity (82.9%) [164]. The identification of the H3-K27M mutation deeply influences patients’ treatment, and the implementation of AI represents a significant step forward in non-invasive, in vivo glioma assessment. 

### 4.7. Ki-67

Ki-67 is a cell antigen reflecting human cell proliferation and whose expression levels are usually low in healthy brain tissues. Malignant tumours often show higher concentrations of Ki-67 levels and worse prognoses. Therefore, it is mandatory to quantify the Ki-67 marker index before glioma treatment [165]. Few studies demonstrated that low ADC values correlate with HGGs and a high expression of Ki67, reflecting the tumour’s high proliferation and malignancy [60,166]. PWI-ASL-derived TBF has been non-univocally linked to Ki-67 levels. Particularly, some authors demonstrated a positive correlation between TBF and Ki-67 [167,168], while others correlated lower TBF values to aggressive glioblastomas based on the hypothesis that hypoxia-driven HIF-1 activation promotes treatment resistance and tissue invasion [169,170,171]. On the other hand, recent literature has focused on AI-based tools to predict Ki-67 status in gliomas with good results. Sun et al. developed a radiomic prediction model based on T1WI and T2WI features that demonstrated an AUC of 0.773 for T2WI in solid gliomas with good sensitivity (81,8%) and specificity (83,3%) [172]. Finally, Su et al. showed that also the radiomic features extracted from multicontrast MRI may effectively demonstrate increased Ki-67 expression [173].

### 4.8. p53

TP53 is a tumour-suppressor gene located on chromosome 17, which is responsible for the production of p53, namely, a transcription regulatory protein in the p53 pathway [174]. Mutation of TP53 causes inactivation and has been related to gliomagenesis, malignant transformation of LGGs, and poor prognosis [9,175]. MRI standard and advanced MRI sequences did not demonstrate sufficient sensitivity and specificity in telling the TP53 status and p53 protein production. Sun et al. developed a radiomic prediction model based on T1WI and T2WI features that demonstrated an AUC of 0.709 for T2WI in solid gliomas’ peritumoral area with optimal sensitivity (100%) and low specificity (40%) [172]. Li et al. developed an ML algorithm to predict p53 pre-operative levels and demonstrated that MR image texture features predict TP53 mutation status in lower-grade gliomas [176].

### 4.9. ATRX

ATRX (alpha thalassemia intellectual disability syndrome X-linked) is a tumour-suppressor gene encoding for a chromatin remodelling and transcriptional regulator protein intercalated in key molecular pathways, such as the regulation of chromatin state, gene expression, and DNA damage repair [177]. Gliomas with ATRX mutation commonly also present IDH mutation and show a better prognosis as compared to ATRX-wild-type IDH-mutant gliomas [177,178]. Moreover, ATRX mutation is commonly associated with increased p53 expression and is almost absent in tumours presenting 1p19q codeletion, which is typical of oligodendrogliomas [179]. Thanks to AI applications to MRI sequences, ATRX mutation status may also be predicted by radiomics features based on the LASSO regression model [180].

## 5. Conclusions

The integration of standard and advanced MRI sequences is paramount to guide glioma differential diagnosis. Moreover, radiomics and radiogenomics models represent a promising frontier in glioma detection and grading, genetic profile definition, prognostication, and pre-surgical planning. Current limitations, such as standardisation challenges, data scarcity, model reproducibility and generalizability, and interpretability concerns, as well as ethical and privacy challenges, remain key issues to address. Future perspectives, rooted in enhanced standardisation and multi-institutional validation, advancements in multi-omics integration, and explainable AI and federated learning, may effectively overcome these challenges and promote efficient AI-based models in glioma management.

## Figures and Tables

**Table 1:** Illustrates a comprehensive, suggested MRI protocol for suspicious incidental low-grade gliomas.

MRI Protocol	
**Scanner**	1.5T or 3T
**Brain Coil**	32- or 64-channel head coil
**Sequences**	
**Pre-contrast**	**Parameters**
Axial TSE T2WI	TR 3000 ms, TE 89 ms, ST 4 mm
(Axial or) 3D FLAIR	TR 4600 ms, TE 328 ms, ST 1 mm—isotropic voxel
Axial T1WI SE (optional)	TR 8600 ms, TE 10 ms, ST 4 mm
SWI	TR 49 ms, TE 40 ms, FA 15°, ST 2 mm
Axial DWI	TR 3150 ms, TE 47 ms, FA 75°, ST 4 mm, b0-500–1000
(32- or) 64-direction axial DTI	TR 8869 ms, TE 73 ms, ST 2.10 mm
Sagittal 3D T1 IR	TR 8.2 ms, TE 3.8 ms, ST 1 mm—isotropic voxel
MRS	SV PRESS (TR 2000 ms, TE 144 ms, voxel 20 × 20 × 20)
**Post-contrast**	**Parameters**
Sagittal 3D T1 IR	TR 8.2 ms, TE 3.8 ms, ST 1 mm—isotropic voxel
Axial T1WI SE (optional)	TR 8600 ms, TE 10 ms, ST 4 mm
PWI	* DSC (TR 1500 ms, TE 40 ms, FA: 60°, ST 4 mm, FOV 24–26 cm, bandwidth 250 kHz—scan time 90 s) * DCE (TR 4.5 ms, TE 1.6 ms, FA 12°, ST 2.20 mm, FOV 24–26 cm, bandwidth 41.67 kHz—scan time 8 min) * ASL (3D-PseudoContinuous ASL: LD 1800 ms, PLD 2025 ms, SI 8; PPS: 512, ST 4.0 mm, FOV 24–26 cm; IPR 3.64–4.53 mm^2^, bandwidth 62.5 kHz, TE 10.9 ms, TR 4840 ms—scan time 4–5 min)—no contrast administration
Task-Based fMRI	The patient usually performs 4 runs of 1 task each: 2 motor tasks and 2 language tasks organized in a box car paradigm (6 baseline and 6 activation periods; 15 s on, 15 s off). The neuroradiologist indicate the motor tasks to the patient, who performs repetitive clenching movements of the hands (in one run) and feet (in the second run). The language tasks consist of silently name objects (in one run) hand actions (in a second run). Stimuli are taken from the object and action naming of the BADA (Battery for the assessment of aphasic disorders). fMRI analysis is performed on the subject’s data using the FMRIB Software Library.

* Alternative sequences MRI (Magnetic Resonance Imaging), WI (weighted Imaging) DWI/ADC (Diffusion WI/Apparent Diffusion Coefficient), DTI (Diffusion Tensor Imaging), MRS (MR Spectroscopy), FLAIR (Fluid-Attenuated Inversion Recovery), SWI (susceptibility WI), IR (inversion recovery), TSE (turbo spin-echo), SE (spin-echo), TR (repetition time), TE (echo time), ST (slice thickness), PRESS (Point RESolved Spectroscopy), SV (single voxel), PWI (Perfusion WI), DSC (Dynamic Susceptibility Contrast), DCE (Dynamic Contrast Enhancement), ASL (Arterial Spin Labelling), fMRI (functional MRI), LD (labelling duration), PLD (post-labelling delay), SI (spiral interleaves), PPS (points per spiral), FOV (field of view), IPR (in-plane resolution).

**Table 2:** Describes the biological role and mutation of glioma genetic markers and the most common MRI changes in the case of gene mutations.

Genetic Biomarker	IDH	EGFR	TERT	MGMT	1p/19q	H3-K27M	Ki-67
**Biological Role**	Regulates angiogenesis	Regulates angiogenesis	Promoter of a component of the enzyme telomerase	Removes the methyl group added to DNA by temozolomide	Typical codeletion of oligodendrogliomas	Biomarker of paediatric diffuse midline gliomas	Reflects human cell proliferation (low values)
**Mutation**	Downregulation of HIF-1α and neoangiogenesis	Amplification favours tumour neoangiogenesis and accelerates proangiogenetic growth factors	Mutation enhances telomerase activity causing alteration of cell longevity and replicative potential	Methylation decreases MGMT promoter activity, improves response to temozolomide and patients’ overall survival	Typical codeletion of oligodendrogliomas	Aggressive growth patterns and resistance to therapy	High values of Ki-67
**PWI**	**DSC:** Decreased rCBV **DCE:** decreased Ktrans, vp, ve **ASL:** increased TBF, nTBG	**DSC:** Increased rCBV **DCE:** increased Ktrans, vp **ASL:** increased CBF	**DSC:** Increased rCBV **DCE:** increased ve	**DSC:** Decreased rCBV **DCE:** decreased ve **ASL:** increased CBF	**DSC:** Increased rCBV in OG3 vs. IDH-mutant gliomas, and decreased rCBV in OG3 vs. IDH-wt gliomas **DCE:** decreased Ktrans and ve as vs. HGGs	_	**ASL:** increased/decreased TBF
**MRS**	Increased 2HG	_	_	_	_	_	_
**CEST—APT**	Lower APT signal	_	_	Lower APT signal	_	_	_
**SWI**	Lower ITSS score, Lower LIV	_	_	Lower ITSS	_	_	_
**ADC**	Increased values	Lower values	_	Higher values	_	Lower values	Lower values
**T2/FLAIR**	T2-FLAIR mismatch	_	_	_	SWITW sign on T2WI	_	_

*IDH (Isocitrate Dehydrogenase), EGFR (Epidermal Growth Factor Receptor), TERT (Telomerase Reverse Transcriptase), MGMT (O6-Methylguanine-DNA methyltransferase), ATRX (Alpha-thalassemia intellectual disability syndrome X-linked), HIF-1α (Hypoxia-Inducible Factor 1-Alpha), TBF (tumor blood flow), nTBF (normalized TBF), DSC (Dynamic Susceptibility Contrast), rCBV (relative Cerebral Blood Volume), CBF (Cerebral Blood Flow), DCE (Dynamic Contrast Enhancement), ASL (Arterial Spin Labelling), MRS (Magnetic Resonance Spectroscopy), 2-hydroxyglutarate, CEST (Chemical Exchange Saturation Transfer), APT (Amide Proton Transfer), SWI (Susceptibility Weighted Image), ITSS (Intratumoural Susceptibility Signals), LIV (Local Image Variance), ADC (apparent diffusion coefficient), FLAIR (Fluid-Attenuated Inversion Recovery), SWITW (wave-like intratumoral-wall), OG3 (oligodendrogliomas grade 3), wt (wild-type), LGG (low-grade glioma), HGG (high-grade glioma).*

## Data Availability

The data are available from the corresponding author, A.R. (Andrea Romano), upon reasonable request.

## Abbreviations

MRI (Magnetic Resonance Imaging), WMTs (white matter tracts), WM (white matter), WI (Weighted Imaging), WHO (World Health Organization), DWI/ADC (Diffusion WI/Apparent Diffusion Coefficient), FLAIR (Fluid-Attenuated Inversion Recovery), SWI (Susceptibility WI), MIP (Minimum Intensity Projections), ITSS (Intratumoural Susceptibility Signals), LIV (Local Image Variance), DTI (Diffusion Tensor Imaging), FA (Fractional Anisotropy), MD (mean diffusivity), NS (number of streamlines), PWI (Perfusion WI), DSC (Dynamic Susceptibility Contrast), rCBV (relative Cerebral Blood Volume), DCE (Dynamic Contrast Enhancement), ASL (Arterial Spin Labelling), fMRI (functional MRI), NSs (number of slices), BR (base resolution), BIR (bolus inversion time), TTP (time-to-peak), MRS (MR Spectroscopy), NAA (N-acetylaspartate), Cho (Choline), Cr (Creatine), mI (myo-Inositol), TR (Repetition Time), TE (Echo Time), IR (Inversion Recovery), ST (Slice Thickness), TSE (Turbo Spin-Echo), SE (Spin-Echo), SV (Single-Voxel); MV (Multi-Voxel), iLGG (incidental Low-Grade Glioma), HGG (High-Grade Glioma), EPI (Echo-Planar Imaging), VOI (Volume of Interest), ROI (Region of Interest), IDH (Isocitrate Dehydrogenase), αKG (α-ketoglutarate) 2HG (2-hydroxyglutarate), EGFR (Epidermal Growth Factor Receptor), TERT (Telomerase Reverse Transcriptase), MGMT (O6-Methylguanine-DNA methyltransferase), ATRX (Alpha-thalassemia intellectual disability syndrome X-linked), DNA (Deoxyribonucleic Acid), AI (Artificial Intelligence), ML (Machine Learning), DL (Deep Learning), LASSO (least absolute shrinkage and selection operator), GBM (glioblastoma), SWITW (wave-like intratumoral wall), BADA (Battery for the assessment of aphasic disorders), LD (labelling duration), PLD (post-labelling delay), SI (spiral interleaves), PPS (points per spiral), FOV (field of view), IPR (in-plane resolution), ISMRM (International Society for Magnetic Resonance in Medicine), 3D pcASL (3D Pseudo Continuous ASL), TBF (tumour blood flow), nTBF (normalized TBF), T1-mVISTA (3D T1 TSE black-blood sequence with sub-millimetre resolution), ROIs (regions of interest), VOIs (volumes of interest), (XAI) Explainable AI, FL (Federated Learning).
